# A gastric ulcer: double trouble

**DOI:** 10.4322/acr.2021.376

**Published:** 2022-04-20

**Authors:** Pauline Verhaegh, Hajo Flink, Alette Daniels-Gooszen, Clément Huysentruyt, Erik Schoon

**Affiliations:** 1 Maastricht University Medical Center, Division Gastroenterology-Hepatology, Department of Internal Medicine, Maastricht, The Netherlands; 2 Catharina Hospital, Catharina Cancer Institute, Department of Gastroenterology and Hepatology, Eindhoven, The Netherlands; 3 Catharina Hospital, Catharina Cancer Institute, Department of Radiology, Eindhoven, The Netherlands; 4 Catharina Hospital, Stichting PAMM, Laboratory of Pathology, Eindhoven, The Netherlands; 5 Maastricht University, GROW School for Oncology and Developmental Biology, Maastricht, The Netherlands

**Keywords:** Sarcina, Helicobacter pylori, Stomach ulcer

To the Editor,

With great interest, we read the article of Marcelino et al.^1^ reporting forty-seven cases of *Sarcina ventriculi*. The article gives an extensive overview of cases describing S. ventriculi appearance, with a sudden increase in cases since 2010. They showed that *S. ventriculi* was identified at all ages, ranging from 1 to 87 years old, but mainly in middle-aged adults. It occurred slightly more often in women (55%). The most common symptoms were epigastric pain (51%) and nausea and vomiting (47%). However, the clinical presentation is heterogeneous, ranging from asymptomatic to life-threatening conditions.^1^ The presence of *S. ventriculi* together with *Helicobacter pylori* 2 or after treatment and eradication of *H. pylori* 3 has been described.

We would like to add another case to the overview provided by Marcelino et al.^1^

**Figure 1:**
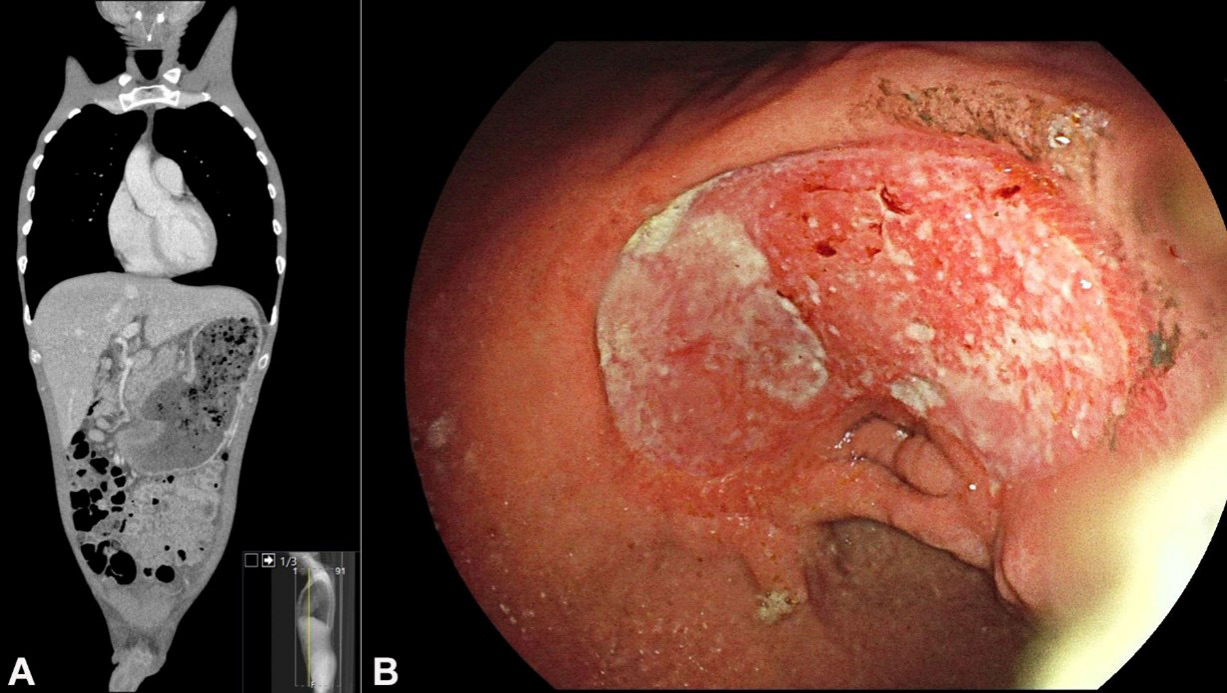
**A–** Thoracoabdominal CT - Coronal plane - showing an abnormal contour of the stomach due to a suspected ulcerating mass, with several enlarged lymph nodes close to the gastrohepatic ligament and around the stomach; **B –** Endoscopic image showing a large ulcer in the antrum of the stomach with a regular and elevated border.

Our patient, a 40-year-old male, was primarily admitted to the oncology department because of nausea and vomiting due to impaired gastric emptying by stenosis. Computed tomography (CT) scan showed signs suspicious for gastric carcinoma with lymphatic metastasis (Figure 1A). Upper GI-endoscopy showed grade-D gastroesophageal reflux disease and a large ulcer in the antrum, with signs suspicious of gastric carcinoma (Figure 1B) and gastric outlet obstruction. Biopsies were taken on three different occasions in the first two weeks of hospitalization.

Histologic examination showed chronic active inflammation, and repeatedly failed to show a malignancy. Also, a needle biopsy of an enlarged lymph node, performed by the radiologist, did not show malignancy. The patient underwent a diagnostic laparoscopy, showing no signs of malignancy. A jejunostomy tube was placed, during this procedure, permitting the patient to be properly fed.

**Figure 2:**
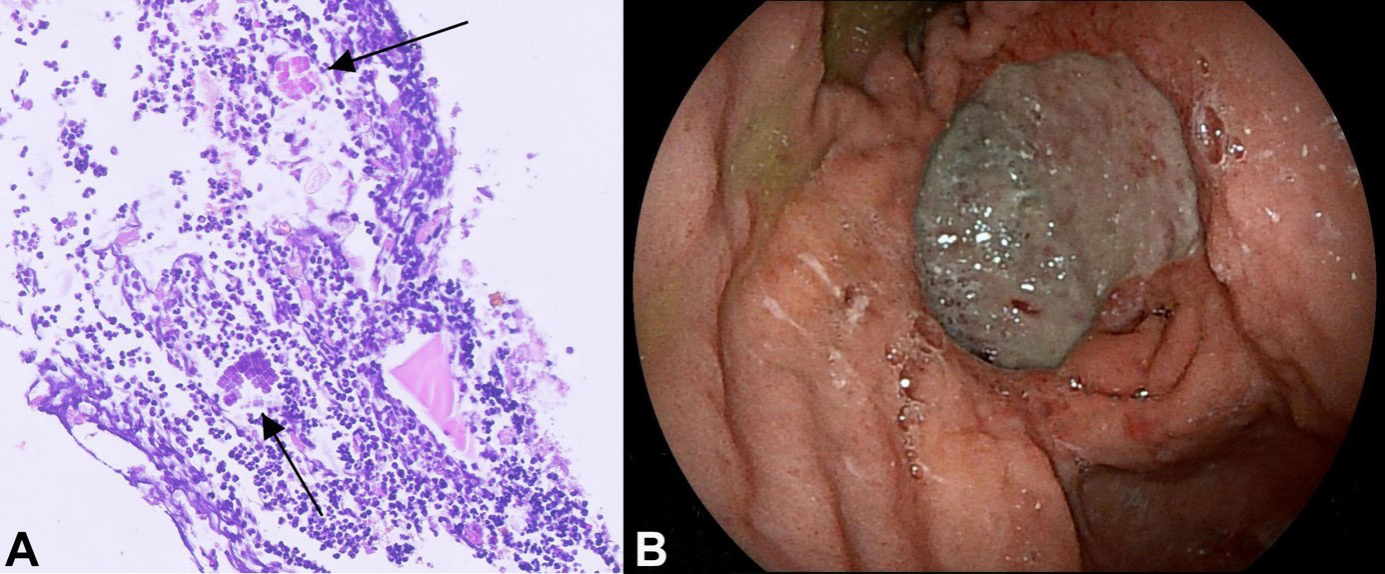
**A –** Photomicrograph of the gastric biopsy showing *S. ventriculi*, the bacteria are typically arranged in cuboids, tetrahedral structures (arrows) (H&E, 10x); **B –** Endoscopic image showing a smaller ulcer in the gastric antrum after treatment for *H. pylori* and *S. ventriculi*.

With these findings, the potential diagnosis that this was ‘just’ an ulcer caused by *H. pylori* became more and more probable, since the biopsies taken during upper GI-endoscopy did show *H. pylori*. Treatment with pantoprazole, amoxicillin and clarithromycin was started and high dose pantoprazole was continued until follow-up endoscopy. Eight weeks later, a follow-up endoscopy was performed, showing a normal esophagus, but the gastric ulcer was still present, albeit smaller in size. Again, biopsies were repeated. This time, the biopsies revealed the presence of *S. ventriculi* (Figure 2A), which might explain the size and severity of the gastric ulcer. Treatment with ciprofloxacin and metronidazole was started. Follow-up endoscopy 8 weeks later showed that the ulcer was still present after treatment (Figure 2B), and biopsies still showed the presence of *S. ventriculi*, and no signs of malignancy. Furthermore, an additional follow-up CT scan of the abdomen was performed, showing no signs of gastric cancer.

There is no established standard treatment for *S. ventriculi*; the most commonly used regime is the combination of ciprofloxacin and metronidazole.^1^ After consultation of a microbiologist, the patient was started on an extended antibiotic treatment of ciprofloxacin and metronidazole for three weeks, in combination with high dose pantoprazole. The patient has not yet been subjected to a subtotal gastrectomy because his condition is improving and he can be properly fed via the jejunostomy tube. Furthermore, questions have been raised about whether performing a subtotal gastrectomy in the presence of *S. ventriculi* might lead to (similar) problems in the future because the bacteria is still present. After finishing the extended treatment, new evaluations will take place.

This case fits in the description given by Marcelino *et al*., with the patient being a middle-aged adult, with symptoms of nausea and vomiting. In this case, *S. ventriculi* was present after the eradication of *H. pylori.*
